# Exploring Staff Implementation of a Self-directed Parenting Intervention for Parents with Mental Health Difficulties

**DOI:** 10.1007/s10597-020-00642-3

**Published:** 2020-05-22

**Authors:** J. Butler, L. Gregg, R. Calam, A. Wittkowski

**Affiliations:** 1 School of Health Sciences, University of Manchester, Manchester, UK; 2 Greater Manchester Mental Health NHS Foundation Trust, Manchester, UK; 3 Division of Psychology and Mental Health, School of Health Sciences, Faculty of Biology, Medicine and Health, Manchester Academic Health Science Centre, The University of Manchester, Zochonis Building, Brunswick Street, Manchester, M13 9PL England, UK

**Keywords:** Parenting, Self-administered, Mental health, Implementation

## Abstract

Parents with mental health difficulties face significant barriers in accessing evidence-based parenting interventions. Self-directed approaches may be a destigmatising, accessible alternative. Evidence has suggested that Triple P Positive Parenting Programme’s self-directed format is as effective as more time- and cost-intensive delivery methods. The aim of the current study was to establish whether staff were able to use this intervention with parents with mental health difficulties and to explore staff experiences of implementation. Triple P self-help workbooks were provided to practitioners across three teams. Data were collected regarding workbook uptake and use. Interviews with staff exploring their experiences of implementation were analysed using thematic analysis. Overall, 41 participants were recruited, of which 12 (29.27%) also consented to interviews. Overall, six practitioners (14.63%) reported that they utilised the workbook. Uptake and utilisation were varied, but practitioners who used the workbook reported positive outcomes. Interviews revealed themes regarding practitioner concerns, views of the intervention and implementation issues. Self-directed Triple remains a promising intervention but its feasibility is dependent on addressing barriers to implementation and facilitating a family-focused approach to meet the needs of these parents and their children.

## Introduction

Parents with mental health difficulties and their children are recognised as a group that face significant and complex challenges (Diggins 2011). Mental health problems have been identified as the leading cause of global disability (Whiteford et al. 2016). Estimates suggest that 68% of women and 57% of men with mental health problems are also parents (Gopfert et al. 2011; Royal College of Psychiatrists 2017). The risks and challenges for these parents and their children have been highlighted (Bee et al. 2014; Schrank et al. 2015). Indeed, children of parents with mental health difficulties are at increased risk of attachment difficulties and social, emotional, behavioural and educational problems (Bee et al. 2014; Manning and Gregoire 2009; Royal College of Psychiatrists 2017), whereas these parents are at higher risk of relapse and rehospitalisation, stigmatisation and social disadvantage than those who are not parents due the additional burdens and stressors they face (Leight et al. 2010; Royal College of Psychiatrists 2011). Parents with mental health difficulties report their difficulty interferes in the relationship they have with their children (van der Ende et al. 2010) because their symptoms can restrict their consistency and availability. Moreover, parental mental health difficulties are frequently present in cases of child maltreatment (Sidebotham et al. 2016).

A number of significant barriers, including the poor integration of mental health and child care services (Stanley et al. 2009), staff feeling ill-equipped to meet the needs of families (Laletas et al. 2017), lack of recognition of family circumstances (Diggins 2011) and reluctance of parents to seek help due to stigma and fear of losing custody (Ackerson 2003; Blegen et al. 2010), means that these families have historically been poorly served by health and social care services.

However, experiencing mental health difficulties does not preclude parents from parenting well (Reupert and Maybery 2011), particularly if they are offered the right support at the right time (Hogg 2013). The lack of provision of effective interventions for parents with mental health difficulties and their children has been identified as a public health concern (Bee et al. 2014; Schrank et al. 2015). A comprehensive evidence synthesis by Bee et al (2014) concluded that evidence for interventions amongst this population is lacking and recommended that rigorous development work is needed to establish interventions that are feasible, acceptable and both clinically and cost-effective.

The economic argument for early intervention as a means of breaking the cycle of disadvantage has been made (Allen 2011; Bauer et al. 2014). Parenting interventions have the potential to provide clinically and cost-effective methods to improve the health and well-being of parents and children (Barlow and Coren 2018; Barrett 2010). Various delivery modalities have been found to be effective including self-administered (Gordon 2000; Markie-Dadds et al. 1999) and web-based programmes (Jones et al. 2017; Sanders et al. 2012). Findings also indicate that self-directed approaches may offer destigmatising, accessible, brief and cost-effective alternative to traditional group-based parenting interventions which parents with mental health difficulties can face significant barriers in accessing (Ackerson 2003; Isobel et al. 2016; Phelan et al. 2013).

The Triple P Programme is a well-established evidence-based parenting programme with a multi-level system of delivery (Sanders et al. 2003; Sanders et al. 2014). A growing body of evidence has suggested that the self-directed format of Triple P (i.e. *Every Parent’s Self-Help Workbook)* (Markie-Dadds and Sanders 2006; Markie-Dadds et al. 1999; Morawska and Sanders 2006) is as effective as more time- and cost-intensive delivery methods (Sanders et al. 2007; Sanders et al. 2000). Emerging evidence has begun to explore the benefits of different formats of Triple P specifically for parents with mental health difficulties, demonstrating improvements in both parent and child outcomes (Jones et al. 2014; Jones et al. 2017; Tsivos et al. 2015, Wolfenden et al. submitted). Self-directed Triple P appears to be an acceptable intervention for parents with mental health difficulties (Wolfenden et al. submitted).

A family-focused approach to mental health care has been adopted internationally, offering a means to meet the needs of parents with mental health difficulties and their children (Foster et al. 2016; Reupert et al. 2015). Local authorities in the UK have attempted to offer a systematic and multiagency approach to early intervention via the provision of Early Help services, which aim to offer support, at the earliest opportunity, to children and families living in difficult circumstances including those affected by parental mental health difficulties (Department for Education 2018; Taylor et al. 2019). The family-focused role of Early Help services provides an opportunity to address the identified barriers to accessing support families with mental health difficulties face, including the poor integration of mental health and child services (Stanley et al. 2009). Thus, the Early Help workforce is likely to be ideally placed to implement a self-directed parenting intervention for parents with mental health difficulties and were therefore identified as appropriate participants in the current study. Existing literature has identified successful implementation of new ways of working is dependent on a range of individual and systematic factors (e.g., availability of workforce training, perceived self-efficacy, perceptions of the intervention; Asgary-Eden and Lee 2012; Sanders et al. 2009).

As no study to date has explored the feasibility of implementing self-directed Triple P for parents with mental health difficulties, our aims were: (1) to establish whether existing front-line staff within Early Help services would use a self-directed parenting intervention (i.e. Triple P *Every Parent’s Self-Help Workbook*) with parents with mental health difficulties and (2) to explore staff experiences of implementation within a family-focused setting.

## Methods

### Study Design

The study was conducted across two phases: In Phase 1, Triple P self-help workbooks were provided to Early Help practitioners and data were collected regarding their uptake and use. In Phase 2, semi-structured interviews were conducted with some staff to explore their perspectives on the feasibility of utilising the Triple P self-help workbook and their experiences of implementation. Full ethical approval was granted by the University of Manchester  Ethics Committee (UREC reference 2018-3173-5877).

### Participants

Participants were Early Help practitioners recruited from three geographical Early Help teams within local authority Children and Family Services in the North West of England, UK. All practitioners were invited to participate in the study. Participants were included if they were proficient in English and currently worked with parents with mental health difficulties. The training and professional backgrounds of these practitioners could be varied; they were not required to have a core health or social care profession.

### Procedure

The primary researcher (JB), met with Early Help team managers from local authority Children and Family Services and provided them with invitation packs, which were distributed to eligible staff. Participants were asked to provide written consent and to complete a demographic questionnaire for contextual information about the sample.

#### Phase 1: Provision of Workbooks and Monitoring of Uptake

Participants were invited to attend a brief information session (45–60 min) that provided information about the Triple P programme and a summary of its evidence base to date. *Every Parent’s Self-Help Workbook,* is a self-directed behavioural parenting intervention, designed to be completed over a 10-week-period to (a) help parents develop positive parenting skills, (b) to increase pro-social child behaviours and (c) to decrease problem behaviours (Markie-Dadds and Sanders 2006; Markie-Dadds et al. 1999). Each practitioner received three Triple P self-help workbooks initially (they were able to request further copies) alongside written instructions (e.g., who the workbooks should be provided to, suggestions for introducing the workbook to parents and how the use of workbooks might be facilitated). Practitioners were asked to use them with parents who (a) had a known diagnosis of a mental health difficulty (or whom they perceived to have mental health difficulties) and (b) had a child or children aged 2–12 years old.

Five weeks after their attendance at the information session, participants were contacted via email to remind them of their involvement in the study. Further email contact was made after 10 weeks asking participants whether they had utilised the Triple P self-help workbooks and how many they had used. Referral and caseload data (i.e. number of referrals to the service, caseload numbers and number of referrals where parental mental health difficulties were identified) were collected from the participating teams.

#### Phase 2: Interviews with Practitioners

Following the 10-week-period, all 41 participants who consented to be contacted were invited to participate in an interview irrespective of whether they had used the workbooks or not. Written consent was obtained prior to commencing the interview. A semi-structured interview schedule was developed and topics included utilisation and views of the workbook, facilitators and barriers encountered and perceived support needs. Interviews, which lasted between 14 and 53 min (average of 39 min, 21 s), were conducted with participants in a private room at the participant’s place of work (the option of an alternative location or via telephone were also offered). Interviews were audio-recorded and transcribed.

### Data Analysis

Thematic analysis, a method for identifying, analysing and reporting patterns or themes, was used to analyse interview data (Braun and Clarke 2006; Creswell 2007). Thematic analysis was used as an essentialist method to report the experiences, meanings and reality of participants in order to explore issues related to the implementation of the intervention (Braun and Clarke 2006; Potter and Wetherell 1987). Braun and Clarke’s (2006) six-stage-process was followed in the present study (familiarisation with the data, generating initial codes, searching for themes, reviewing themes, defining and naming themes**)**. Data were coded inductively, rather than utilising a predefined framework to ensure the analysis was data-driven. NVivo software (QSR International Pty Ltd. Version 12, 2018) was used to organise and code the transcripts.

### Reflexivity Statement and Validity Checking

The primary researcher JB was a trainee clinical psychologist with experience of delivering evidence-based parenting programmes. LG, RC, and AW were experts in mental health and/or parenting research. AW has been trained in Triple P. However, LG was not trained in Triple P, which allowed the analysis to be conducted from a relatively non-expert standpoint, but the potential for bias is acknowledged. Steps were taken to ensure the analysis process was sufficiently rigorous and the potential for bias was minimised including coding data inductively and processes of validity checking involving multiple authors.

The primary researcher led the analysis with support from the research team. A selection of codes was reviewed by authors AW  and LG  to ensure the interpretation of the primary researcher was derived from the data. All authors reviewed and agreed the thematic structure.

## Results

### Phase 1: Uptake of the Workbooks

Overall, 41 participants were recruited to the study and a subset of 12 practitioners (29.27%) took part in semi-structured interviews. In terms of sample characteristics, demographic information was available for only 32 (78.05%) of the 41 participants. The remaining participants chose not to complete the questionnaire. Participants were mostly white females who had extensive experience working with parents and families (see Table 1 for further details).

**Table 1:** Demographic characteristics of the sample

	N (%)
Gender	
Female	28 (68.29%)
Male	4 (9.36%)
Unknown	9 (21.95%)
Age (years)	46.33 (*SD* 7.88; range 23–56)
Ethnicity	
White	25 (60.98%)
Black/African/Caribbean	4 (9.76)
Asian	1 (2.38%)
Prefer not to say	2 (4.88%)
Unknown	9 (21.95%)
Highest education level	
Secondary school	10 (24.39%)
Additional vocational	10 (24.39%)
Undergraduate degree	5 (12.20%)
Unknown	18 (39.02%)
Average time working in current service (years)	7 years 3 months (range 3 months–37 years 2 months)
Years’ experience working with parents and families	
1–5 years	2 (4.49%)
10+ years	28 (68.29%)
Unknown	11 (26.83%)

**Fig. 1:**
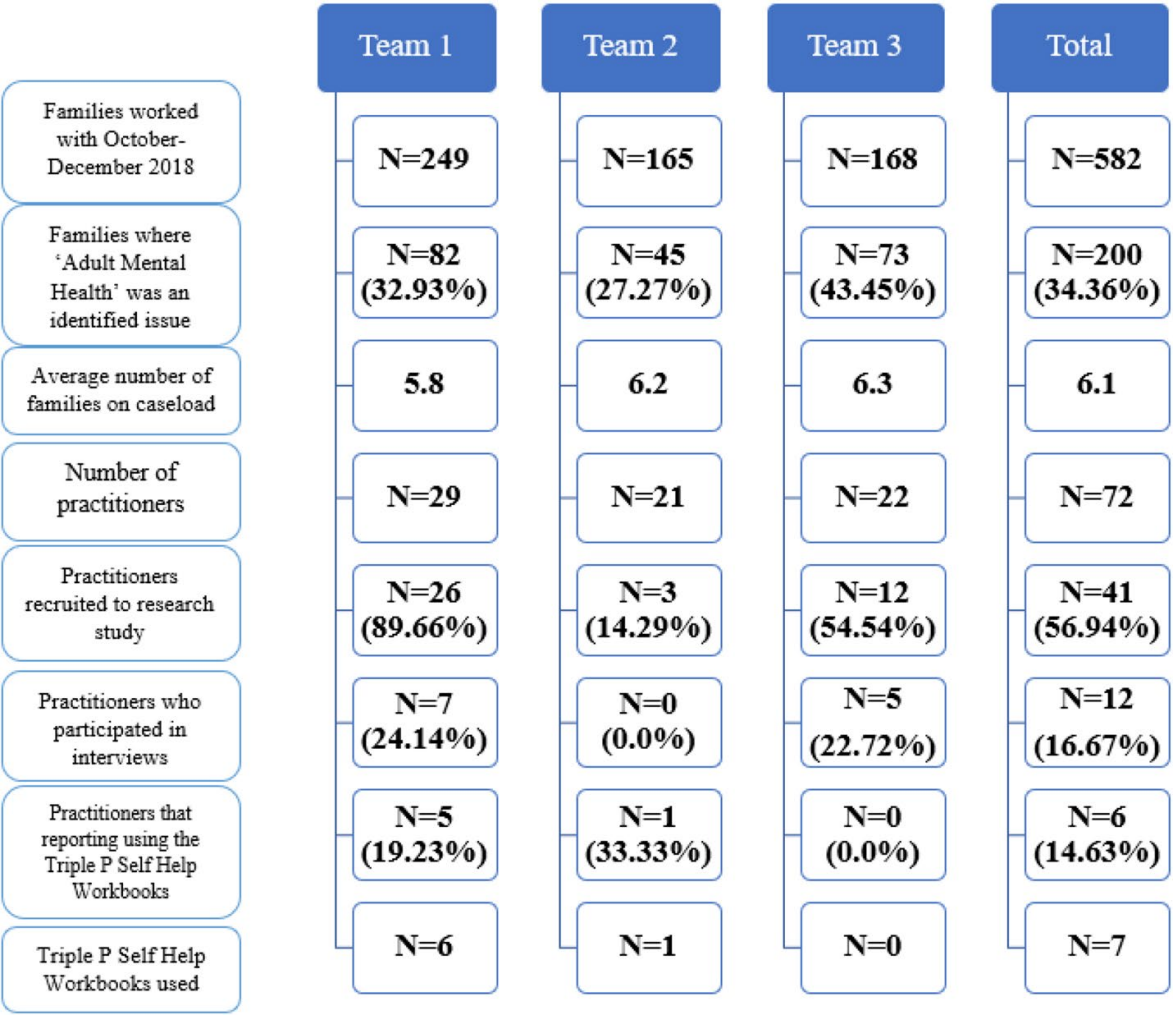
Flowchart of utilisation of the Triple P self-help workbooks

During the research period 72 practitioners worked across the three participating teams (Team 1: n = 29, Team 2: n = 21 and Team 3: n = 22); 41 of those 72 practitioners (56.94%) agreed to participate in the study. Overall, six practitioners (15%) reported that they utilised the Triple P self-help workbook, 25 practitioners (61%) did not utilise the workbook and ten (24%) failed to respond. It was notable that practitioner uptake, and use of the workbook, varied significantly across the three teams. The reported incidence of parental mental health difficulties amongst families that practitioners worked with and the average caseload during the research period also varied. Figure 1 illustrates the utilisation of the Triple P self-help workbooks across each of the three participating teams. It also shows the number of families that teams worked with during the research period (October–December 2018), the proportion in which parental mental health difficulties were identified and details of the workbook uptake and its utilisation.

### Phase 2: Interviews with Practitioners

#### Sample Characteristics of Interview Participants

All 41 practitioners who participated in the study were invited to participate in interviews. A subset of 12 practitioners agreed to take part in interviews. Only practitioners from Team 1 and Team 3 consented to be interviewed (n = 7 and n = 5 respectively). Ten women and two men were interviewed. Four of the practitioners interviewed had attempted to utilise the Triple P self-help workbook and eight had not. Most practitioners (n = 10) had previous experience of evidence-based parenting interventions. The four practitioners that reported utilising the workbook provided it to seven parents between them: six mothers and one father. The seven parents were reported to have had a range of diagnosed mental health difficulties (including anxiety, depression, personality disorder and bipolar disorder). Practitioners reported that some parents they provided the workbooks to experienced additional difficulties including a specific learning difficulty, fibromyalgia and parental drug use. The children of these families ranged from five to 15 years of age.

#### Thematic Analysis

Three main themes and eight subthemes were identified. Table 2 provides an overview of the three main themes and eight sub-themes.

**Table 2:** Overview of main themes and subthemes

Theme 1: practitioner concerns	Additional quotes for Theme 1	Theme 2: practitioner views of the intervention	Additional quotes for Theme 2	Theme 3: implementation issues	Additional quotes for Theme 3
1.1 Parents’ capacity: The impact of parents’ mental health difficulties; Parental literacy	“They might wake up low in mood, they don’t want to get out of bed, they don’t want to talk to people, can’t focus, don’t want to read or you know…I mean some with mental health [difficulties] they can’t even get out of bed to brush their hair so…I don’t think they’re going to pick up a book…” (Participant#4, used one workbook) “…[because] a lot of our families, they might have not had a, a very good childhood or, you know, education, so, as soon as they see things like that, they go, oh my god, you know, I can’t do it, and sometimes it’s embarrassing if they don’t, they can, if they say, no I can’t, you know, I can’t read or, or write, that makes them feel very vulnerable.” (Participant #5, did not use the workbook	2.1 Accessibility of the workbook and suggestions for improvement: Cultural and language barriers; Improving accessibility; Use of technology	[No additional quotes]	3.1 Challenges and identified support needs: Training needs; Managing workload	“I’m just thinking, do you think if we done it this way, do you think that’d help or would it affect their mental health worse, ‘cos I’m not a mental health worker, so I wouldn’t know. So sometimes with the trial and error, I wouldn’t wanna kick anything off, so I’d be very reserved thinking, right, I better not do that.” (Participant #12, used two workbooks) “I think….the amount of cases that we’ve got and the level that the cases are at is really hard for…you to be able to do positive work like this consistently because of everything else that you’re dealing with and all the other families that you’ve got to see and everything that’s going on for them.” (Participant #9, did not use the workbook)
1.2 The challenge of meeting parents’ needs A reluctance to engage Establishing priorities: finding the ‘right time’ Meeting unmet needs	“It depends on what, where that parent is on that day, she’s having a really, really rubbish day, she’s not gonna think twice about looking in that book and thinking, I’m gonna do that today, ‘cos it just wouldn’t happen.” (Participant #12, used two workbooks) “It wasn’t at the right time, and I think working with families with mental health is really difficult to find that right time, because it’s, you have to be, somewhere sort of in the middle of, of being stable and able to, to work with you.” (Participant #1, did not use the workbook) “…the mental health workers I know are few and they’re stretched to the limit.” (Participant #11, did not use the workbook) “A lot of agencies, they think we can fix everything, because nobody really knows what we do, so they think, you know you’ve got this hat and you, you know you can, with the hat you can do everything.” (Participant #5, did not use the workbook)	2.1 Positive views of the intervention: Accessible; Value of self-directed intervention: Flexibility, independence, destigmatising, timely	“…We can do the work together, and it not being a stranger as well, then that’s a really good thing about the book that, you’ve built that relationship with that family, so you’re not just sending them off to a course, to somebody that they don’t know, and you know, whoever that lives down the road is going as well, it’s, you know, […] no one has to know that you’re doing the parenting stuff with them, you can kind of just go into the homes and just do it, a little bit at a time and at the parents’ pace, rather than them being like, oh god, I don’t understand, or, you know, sometimes they don’t understand, you know, what the, erm, you know what the facilitator’s saying, or it’s things like that, and don’t wanna be embarrassed, whereas that, if they’ve got a relationship with you, they can go, I don’t have a clue what you’re talking about, or, you know, this is what that, you know, what does that mean, and it, it makes it easier that way.” (Participant #5, did not use the workbook)	3.2 Implementation facilitators: Practical and relational aspects	“I think for me the biggest part of it is the relationship building and think for particularly for families that have got mental health problems and that they’ve got those problems for whatever reason erm…that’s really really important. So I just find that that finding your feet with that and finding that kind of common ground with the families is vital for me to do anything really. Not just parenting but anything.” (Participant #9, did not use the workbook) “…if they don’t, they don’t tell you and you don’t build up that relationship you’re not to know, so like I said about being open and honest with him, I’m not here to judge.” (Participant #6, used one workbook)
		2.2 Positive outcomes: Changes in parental behaviour; Improvements in child behaviour; Improvements in the parent–child relationship: Improved parental confidence; Workbook empowering parents	“…the beauty of it all came to a point where I could see the excitement you know like she was accomplishing something […] and things were working for her and she’s really come out of her shell yeah it was nice to see…because she rang me one time [X] I feel so…wouldn’t say the word empowered but she felt confident with her parenting which at first she was very self-doubting.” (Participant #2, used one workbook) “She was sceptical at, sceptical about doing it at first, she was saying, oh, do we have to do this, we have the meetings and I’ve got him to deal with, but yeah, it works, it did work.” (Participant #12, used two workbooks)		
		2.3. Preference for other delivery formats: Added value of group-based interventions	“…I feel that if when I’ve gone through part of the course with them in the home if they’ve got mental health [difficulties] I find that they don’t focus very well. And I find they do much better in a group.” (Participant #4, used one workbook)		

#### Theme 1: Practitioner Concerns

##### Parents’ Capacity

Practitioners expressed concerns about parents’ capacity to utilise any self-directed intervention due to the impact of their mental health difficulties and literacy issues. The impact of parents’ mental health difficulties was frequently cited as a significant barrier to engaging in such an intervention:

Yeah it…there’s quite…it’s quite a lot of reading and erm….erm….for them to be able to have the concentration to sit down and read a book I don’t think is doable for the families well certainly for the families that I work with. (Participant#9, did not use the workbook).

Parental literacy problems were the most frequently cited barrier to engagement. Practitioners identified that parents may be reluctant to acknowledge such difficulties, making it more challenging to ensure adequate support is offered to overcome this barrier:

A lot of the parents we work with they like…quite a lot are not too great at reading…they’re not educated. (Participant #4, used one workbook)

##### The Challenge of Meeting Parents’ Needs

The perceived complexity associated with trying to meet the needs of these families was highlighted and included a perceived reluctance of parents to engage, difficulties in establishing priorities and finding the right time for any parenting intervention, practitioners attempting to meet unmet needs and other additional barriers.

Practitioners also described the difficulties they encountered in trying to engage these parents in any parenting work. They cited difficulty in committing to parenting interventions, variable mood and intermittent engagement as particular challenges:

I think when you’re working with families with multiple problems they can so easily disengage so even if you start a parenting programme with them particularly with mental health they could just disengage a whole range of different reasons […] but yeah engagement is a big problem or barrier. (Participant #7, did not use the workbook)

Practitioners reported that the parents they worked with who were experiencing mental health difficulties commonly felt inadequate as parents:

…when she was parenting she didn’t feel that she was adequate she always had this self-doubt about herself. (Participant#2, used one workbook)

This in turn made it difficult for some practitioners to offer the intervention without leaving parents feeling they had been criticised or judged a ‘bad parent’:

They take it as […] a form of that I’m not doing well. You don’t think I’m that good then, so you have to be very, very careful how you tread with some families (Participant#6, used one workbook)

When working with families with mental health difficulties, practitioners commonly identified establishing priorities and finding the ‘right time’ for a parenting intervention as a challenge. Practitioners shared a sense that often there is “*so much going on”* (Participant #5, did not use the workbook) for families, their perception was that the parent would not be ready to engage with a parenting intervention:

…I think it was more a case of them not being ready for it…one family in particular it was from one crisis just to another really…they wouldn’t have been able to commit to a parenting course erm…there were too many urgent needs really before they could think about parenting. (Participant #7, did not use the workbook)

Practitioners described often finding themselves needing to ensure families’ basic needs were met, making it difficult to prioritise any intervention work. Addressing safeguarding concerns, issues of domestic violence, homelessness, financial problems and access to sufficient food and electricity were cited as examples. Practitioners identified wider concerns about service provision which left them attempting to meet other unmet needs, resulting from a lack of resource in mental health services:

…so, it’s having, it’s having the mental health worker there to, you know, kind of support your work, but they don’t have the capacity and we don’t have the capacity either to kind of do that, so it is, it, it’s more about higher up people, funding and all that sort of stuff. (Participant #5, did not use the workbook)

Practitioners described their attempts to facilitate parents’ access to mental health services or medication. Given the family-focussed multiagency approach of the Early Help workforce, practitioners spoke of their frustration that at times other agencies did not understand the demands of their role and expected them to ‘fix all’. Practitioners also identified other additional challenges to meeting parents’ needs and barriers to utilising any intervention that relied on parents’ own volition. These included a chaotic environment in the family home, parental drug or alcohol misuse and the parent locating the problem within the child.

#### Theme 2: Practitioner Views of the Intervention

##### Accessibility of the Workbook and Suggestions for Improvement

Some practitioners expressed concerns that the Triple P self-help workbook might be inaccessible for parents with mental health difficulties in its current format. They also identified cultural and language barriers to utilising the workbook, in particular, that the workbook only depicted white parents.

Practitioners made several suggestions to improve the accessibility of the workbook including the provision of a leaflet to introduce the workbook for parents, the availability of the workbook in other languages and the option to provide shorter sections of the workbook to avoid overwhelming parents. Practitioners commonly identified that whilst they valued the information included in the workbook, its accessibility could be improved by reducing the amount of text and presenting information visually or using pictures:

…my initial reading was it’s fantastic […] but I think the whole layout of the book is too… you look at it and you’re not even going to read it because I think it needs to be much simpler, less text, maybe some pictorial stuff in but and not so much on the page. (Participant #11, did not use the workbook)

Moreover, the use of technology (e.g., the provision of audio, video or online materials) to supplement and improve the accessibility of the workbook was also suggested.

##### Positive Views of the Intervention

Participants reported positive views of the intervention’s content, mode of delivery, and its potential future utility amongst parents experiencing mental health difficulties, finding it to be accessible in its current format:

You see, me personally, I think it’s quite, it’s not, it’s not, erm, what’s the word, it’s not full of jargon, it is quite family based, orientated, I think so, that’s my, my interpretation of it. (Participant #6, used one workbook)

I think it from what I could see it was quite easy to follow and I think it was quite user-friendly for parents to use so that was a positive thing. (Participant #7, did not use the workbook)

Some practitioners identified the value of a self-directed intervention as an alternative to group-based delivery in offering a greater level of flexibility and independence for parents, avoiding the stigma or isolation parents with mental health difficulties could feel in a group setting and the potential of offering a timelier intervention in the context of an existing parent-practitioner relationship (see Table 2).

##### Positive Outcomes

Practitioners that made use of the Triple P self-help workbook reported a range of positive outcomes for families including changes in parental behaviour, such as implementing appropriate boundaries for their children and remaining calm, improvements in child behaviour and improvements in the parent–child relationship:

…so it was really nice to see, and at the end of it, we’d finished the book, we’d gone through the book, and their relationship now is a lot better than it was. (Participant #12, used two workbooks)

Practitioners also described a significant improvement in parental confidence and an increased sense of self efficacy: “*there had to be more to life than this and I can do something about this”* (Participant #12, used two workbooks). Practitioners cited examples of the workbook supporting parents to feel empowered to make changes and address parenting challenges independently:

Yeah, it empowered her a lot more than, before, because before she’d phone me, and I’d go round and try and sort it out between ‘em, whereas now she’s doing that for herself. (Participant #12, used two workbooks)

Such positive outcomes were often despite parents being initially sceptical or pessimistic about using the workbook: *“Yeah she said some of the exercises seemed pointless but when she put it into practice it did help.”* (Participant #2, used one workbook).

##### Preference for Other Delivery Formats

In contrast, other practitioners expressed a preference for other modes of delivery over this type of self-directed intervention. In particular, practitioners reported the added value of group-based parenting interventions:

…working with a group I just think they would benefit so much more than just trying to do it on their own […] I also think just the whole group situation just helps them to learn a lot more than they would if they were just doing a book on their own yeah. (Participant #7, did not use the workbook)

#### Theme 3: Implementation Issues

##### Challenges and Identified Support Needs

Practitioners identified several implementation challenges to using and implementing the Triple P self-help workbook for parents with mental health difficulties, including systemic challenges of managing workload and ensuring adequate training. The majority of practitioners identified that they felt ill-equipped to meet the needs of parents with mental health difficulties and they lacked training:

… I think you need to this is more general not just the workbook it’s just about having an understanding of mental health problems and how it affects people which they are there is training for that erm…but you know as an interventions worker you do need to have that understanding of how mental health affects people. (Participant #7, did not use the workbook)

This can be considered in the context of practitioners feeling stretched to meet needs they perceived to be unmet, as a result of a lack of resource in mental health services (see subtheme 1.2), whilst recognising “*I’m not a mental health worker”* (Participant #12, used two workbooks).

Whilst one practitioner reported that the brief information session provided was sufficient, most practitioners reported they would require further training to feel confident enough to make use of the workbooks. Practitioners made suggestions for supporting implementation through the provision of an accessible summary of the workbook for practitioners, telephone support, the opportunity to come together with other practitioners using the workbook and to engage in joint-working with mental health practitioners. The value of clinical supervision was also highlighted in supporting practitioners to manage the emotional demands of their work with families. Finally, practitioners also identified the challenges of managing their workload and having sufficient time to make use of the workbook with families.

##### Implementation Facilitators

Practitioners highlighted a number of practical and relational aspects perceived to be important in facilitating implementation of the workbook. Practitioners reported offering support to help parents overcome literacy problems or memory difficulties, establishing clear expectations to reduce parental anxiety about using the workbook, offering reassurance and normalising the challenges faced by parents, encouraging parents to prioritise and persist with using the workbook and allowing parents to identify an area of focus:

In the beginning it was hard because it was like for her going back to school then I said to her just relax because she was very anxious I said just relax and I just said to her pick what exercises you want to do and what pages would be easy to read and we’ll go through it together and that’s what we did… (Participant #2, used one workbook)

Practitioners found it helpful to highlight to parents the potential benefits of engaging with the workbook. The importance of an environment free from distractions to facilitate the use of the workbook, the value of modelling strategies to parents and the need to adapt strategies to find what works for individual families was highlighted by practitioners. A number of practitioners also spoke of the value of children being aware and involved in the intervention:

I think…what helped her as well was that her daughter could see that she was reading something about parenting…because her daughter was saying for example I’ve never seen my mum read… (Participant #2, used one workbook)

Practitioners spoke of the importance of building a trusting relationship with parents experiencing mental health difficulties. Demonstrating a supportive and non-judgemental stance was seen as essential to establishing an effective working relationship in which parents felt able to be open about their difficulties*.* In addition, practitioners identified the importance of investing in a family, treating parents as individuals, instilling hope, empathising with the difficulties parents face and drawing upon personal experiences of using strategies effectively in order to connect with a parent:

So I do think it is quite hard but then when you talk to them about, like I say I always use, I used the book but I also say well I tried this as well you know so they feel maybe a bit more connected and that you’re not criticising or judging it makes it a little bit easier… (Participant #3, did not use the workbook)

## Discussion

This is the first study to explore the implementation of self-directed Triple P for parents with mental health difficulties. The findings revealed that whilst the reported incidence of parental mental health difficulties was high amongst families that practitioners worked with, overall only 14.63% of practitioners that took part in the study provided the Triple P self-help workbook to a parent during the 3-month research period. However, an exploration of practitioners’ experiences of implementation revealed their concerns regarding the accessibility of the workbook, their views of the intervention and barriers to implementation. Findings indicated the utility of self-directed Triple P for parents with mental health difficulties remains promising: when the workbook was used, practitioners identified the significant benefits that parents derived.

In order to provide real-world context and strengthen validity, practitioners were recruited across three geographical teams, revealing significant variations in uptake and utilisation of the workbook. It is interesting to note that no Triple P self-help workbooks were provided to parents in the team (Team 2) in which the incidence of parental mental health difficulties and practitioner caseloads were highest. Uptake and utilisation of the workbooks was highest in the team that had the lowest average caseload. The fit of the programme with current demands of a practitioner’s role and workload has previously been identified as an important factor in implementation (Sanders et al. 2009; Shapiro et al. 2012). Moreover, practitioner recruitment via team managers is likely to have had a significant impact on the variation in uptake across teams. Existing literature has emphasised that evidence-based parenting programmes are more likely to be implemented when there is workplace support for the initiative (Asgary-Eden and Lee 2012; Sanders et al. 2009).

Qualitative interviews with a subset of practitioners identified clear additional reasons why they did not utilise an additional parenting resource when one was offered to them. Many practitioners expressed their future intention to use the workbook. These findings contribute to the identification of barriers to and facilitators of engaging parents and implementing evidence-based parenting programmes (Asgary-Eden and Lee 2012; Koerting et al. 2013; Shapiro et al. 2012). In the current study, practitioners’ perceptions regarding the capacity of parents with mental health difficulties and the accessibility Triple P self-help workbook were identified as barriers to utilisation. As previously identified in the wider literature (Holloway and Wheeler 2015; Lee 2005), our study also confirmed that practitioners acted as gatekeepers, making a judgement on behalf of a parent about the appropriateness of an intervention. Practitioners are perhaps more likely to make a decision without exploring this with parents due to concerns about parents feeling criticised or judged as a ‘bad parent’, as highlighted in the current study. This may be particularly the case in the context of feelings of parental inadequacy amongst parents with mental health difficulties, and practitioner concerns about the impact such feelings may have on the parent-practitioner relationship (Tchernegovski et al. 2017). Whilst practitioners’ concerns are not surprising, given the stigma associated with parenting with a mental health difficulty and the identified barriers to seeking help (Ackerson 2003; Beresford et al. 2008; Blegen et al. 2010), gatekeeping may serve as an additional barrier for parents with mental health difficulties in accessing evidence-based parenting interventions.

The present study highlights additional practical and relational factors which are likely to facilitate implementation of a self-directed parenting intervention for parents with mental health difficulties. The importance of practitioners employing a supportive and non-judgemental approach to develop a trusting relationship with parents prior to introducing the intervention is emphasised again, as has been previously established (Barlow and Stewart-Brown 2001; Garcia et al. 2018; Butler et al. 2020). The present study ascertains practitioners’ support and training needs in relation to parental mental health as has been previously identified (Laletas et al. 2017). The combination of increasing worker skills and strengthening systems and structures are likely to be essential to successful implementation (Austin and Ciaassen 2008). Ensuring that practitioners feel sufficiently confident and the availability of ongoing post-training support are known to be important factors in influencing the implementation of evidence-based parenting interventions (Asgary-Eden and Lee 2012; Shapiro et al. 2012). Moreover, whilst the workbook is designed to be a self-directed intervention the potential value of a facilitator to support parents in their use of the workbook is indicated. This is in line with previous work which has found that self-directed interventions are more effective if offered in conjunction with minimal facilitation (e.g., brief telephone calls) compared to no assistance (Morawska and Sanders 2006).

In the current study, practitioners identified the value of a self-directed intervention as an alternative to group-based delivery, offering flexibility, promoting independence and avoiding the stigma or isolation parents with mental health difficulties may feel in a group setting. Previous work has also suggested that self-directed approaches may offer a destigmatising, accessible and cost-effective alternative (Sanders et al. 2012, 2007). The acceptability of self-directed interventions for parents has also been demonstrated (Ogg and Carlson 2009; Stewart and Carlson 2010; Thomson and Carlson 2017) and evidence of acceptability, specifically for parents with mental health difficulties, is emerging (Wolfenden et al. submitted). However, parenting interventions are traditionally delivered in a group-format and parents identified the value of shared learning (see review by Butler et al. 2020). It is therefore not surprising that some practitioners expressed a preference for group-based parenting interventions due to perceived added value for parents with mental health difficulties. Indeed, group parenting programmes specifically for parents with mental health difficulties have found combining a group-format and home-based delivery to be effective (Coates et al. 2017; Isobel et al. 2016; Phelan et al. 2013). In line with Triple P’s implementation framework which emphasises the principles of self-regulation and minimal sufficiency (McWilliam et al. 2016), offering a choice of interventions of varying intensity to suit the needs of families is likely to be beneficial (Sanders et al. 2007).

In the present study, encouraging outcomes were seen amongst families when the Triple P self-help workbook was used. In particular, practitioners described parents being empowered to bring about change, known to be a central tenant of effective parenting interventions. These findings fit with a growing body of evidence demonstrating that self-directed parenting interventions show promise as an alternative to group-based interventions (Markie-Dadds and Sanders 2006; Morawska and Sanders 2006; Ogg and Carlson, 2009; Sanders et al. 2007; Thomson and Carlson 2017).

The provision of self-directed parenting interventions show promise for parents with mental health difficulties. Identified barriers to implementation are modifiable, although further work is needed to establish the feasibility of implementing a self-directed approach on a larger scale. It seems likely that practitioner beliefs and attitudes regarding parents’ capacity to use a self-directed parenting intervention contributed to the low rates of uptake and utilisation in the current study. Adopting a problem-solving and formulation-based approach to working with families is likely to support practitioners to make informed decisions about the ‘right time’ for a parenting intervention and to consider it an integral component of recovery orientated work for parents with mental health difficulties (Reupert et al. 2017). A family-focused approach to mental healthcare is likely to require further training for practitioners and collaborative working between services to ensure family’s needs are met (Foster et al. 2016; Maybery and Reupert 2009). Tailored professional training to ensure practitioners are equipped to facilitate open and transparent conversations about parenting is a clear priority (Laletas et al. 2017; Tchernegovski et al. 2017).

Future development of the Triple P self-help workbook could consider practitioners’ suggestions to improve its accessibility, including reducing the amount of text and presenting information visually, ensuring the workbook is representative of parents from diverse cultural backgrounds and the availability of the workbook in other languages and varying formats. Researchers have identified the importance of cultural sensitivity in the delivery of parenting programmes (Mejia et al. 2016; Owens et al. 2008) and alternative formats of Triple P have already been adapted to improve cultural fit (Houlding et al. 2012).

The successful application of evidence-based programmes is contingent not only on effectiveness but how it is implemented and sustained (Fixsen et al. 2005). Findings of the current study emphasise the importance of addressing organisational issues, such as practitioner workload and training needs, if adoption of the intervention is to be successful. This corroborates previous findings that suggest organisational climate and workplace support, predict utilisation of evidence-based parenting programmes (Asgary-Eden and Lee 2012; Sanders et al. 2009). Perceived benefit of the intervention for families is known to predict implementation of evidence-based parenting programmes (Shapiro et al. 2012) and therefore future research efforts should aim to build on the emerging evidence outlined.

### Strengths and Limitations

The collection of interview data from practitioners, who did or did not use the workbook, in addition to recording uptake of the workbook allowed for a greater exploration of the barriers to implementing this self-directed parenting intervention and provides some indication as to how these barriers may be overcome. Whilst the 12 practitioners who participated in interviews represent a small sample size, this is in line with recommendations and was sufficient to ensure data saturation (Guest et al. 2006). Interviews with practitioners from the team with the lowest uptake may have provided valuable additional insights. Moreover, missing demographic data made it difficult to establish whether practitioners who participated in interviews were representative of the wider sample.

Due to study constraints, monitoring uptake of the workbooks had to be restricted to a 3-month-period. Given that practitioners reported having to support families in crisis, it would have been advisable to monitor uptake over a longer period. The short research period may in part have accounted for the low levels of utilisation reported as many practitioners expressed their future intention to use the workbook. It should also be noted, self-directed Triple P does have a video series, but it was not possible to provide practitioners or parents with access to this in the current study. Despite these limitations, conducting the study across three different geographical teams with existing front-line staff, provided a real-world context to explore challenges to implementation.

## Conclusion

The present study offers an exploration of practitioner perspectives and experiences in relation to the implementation of a self-directed parenting intervention for parents with mental health difficulties. Whilst findings indicate uptake and utilisation was varied, valuable insights into practitioner concerns, support needs and organisational implementation issues suggested that a self-directed parenting intervention has potential utility as a flexible and cost-effective approach for services supporting parents with mental health difficulties. The feasibility of this approach is dependent on addressing identified barriers to implementation and facilitating a family-focused and formulation-based approach to meet the needs of parents with mental health difficulties and their children.
